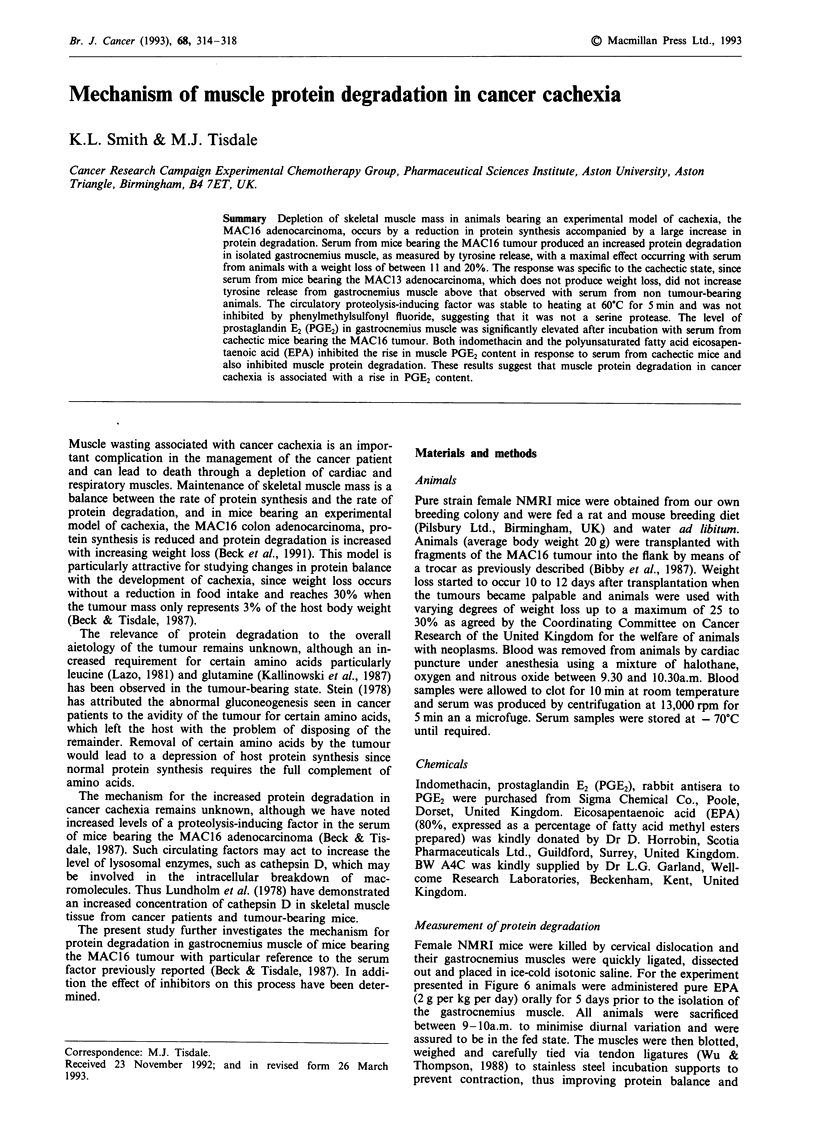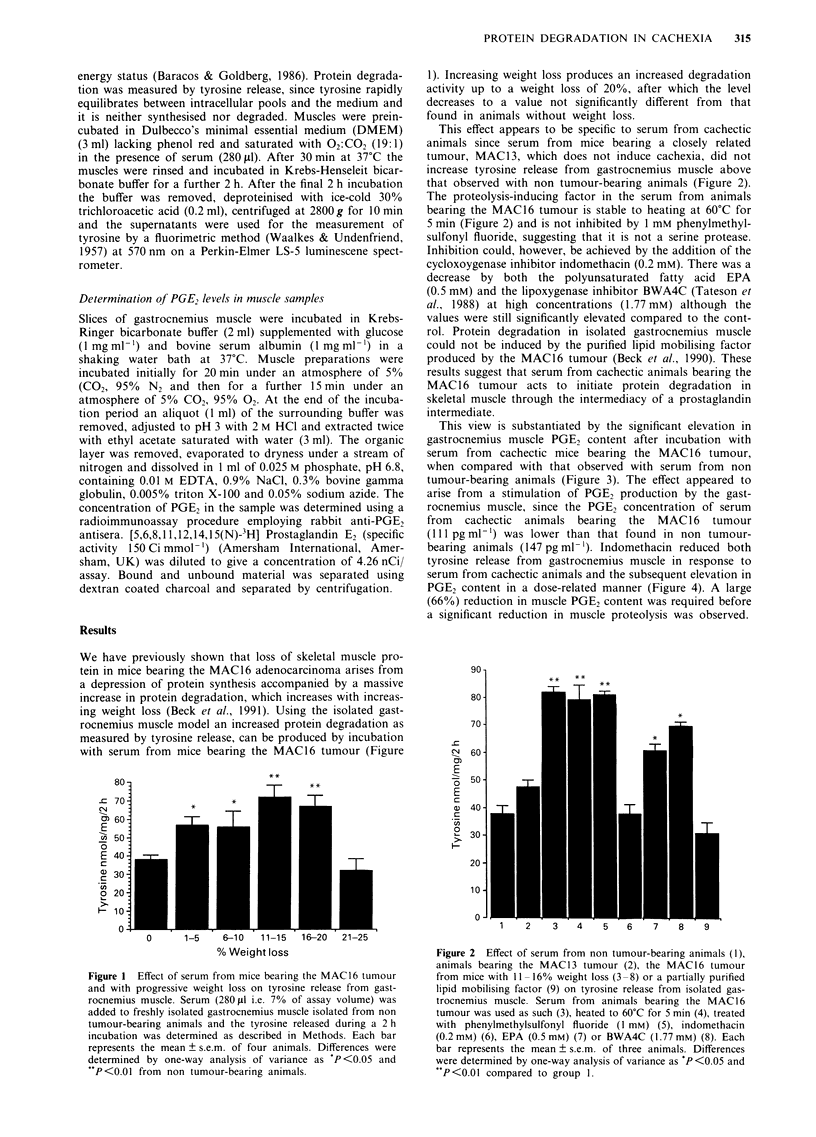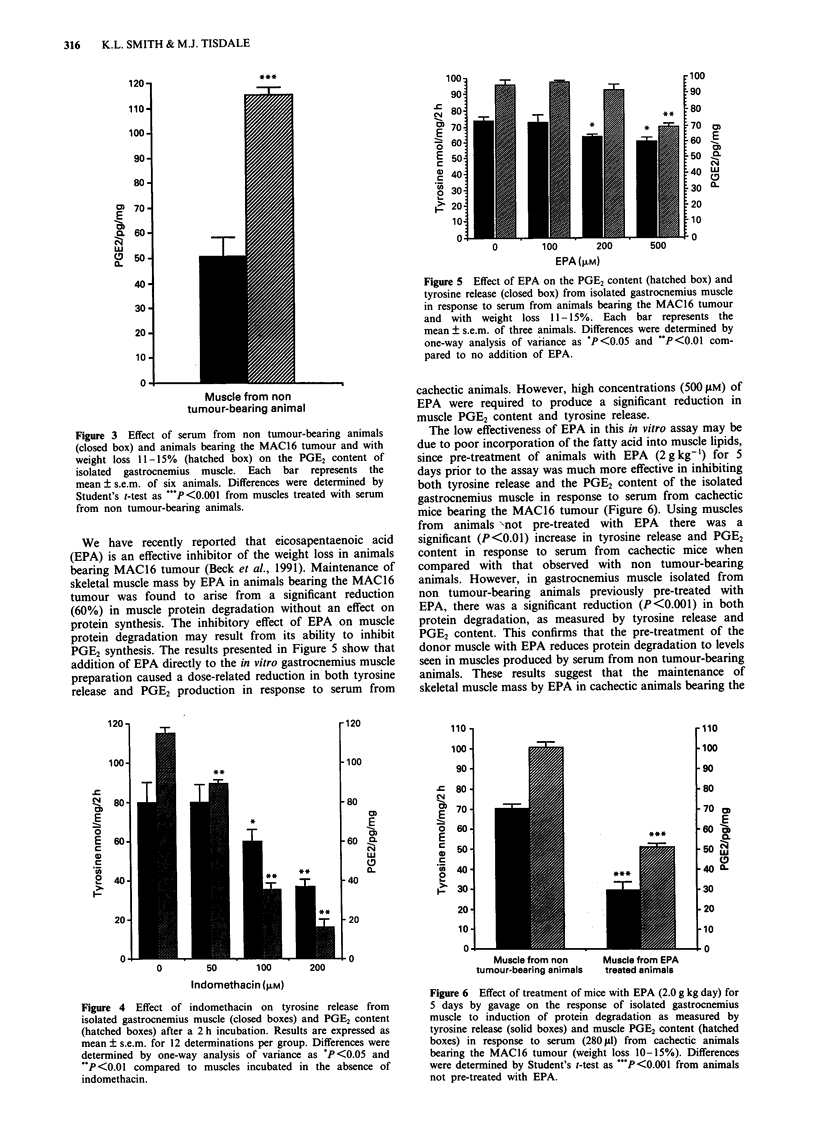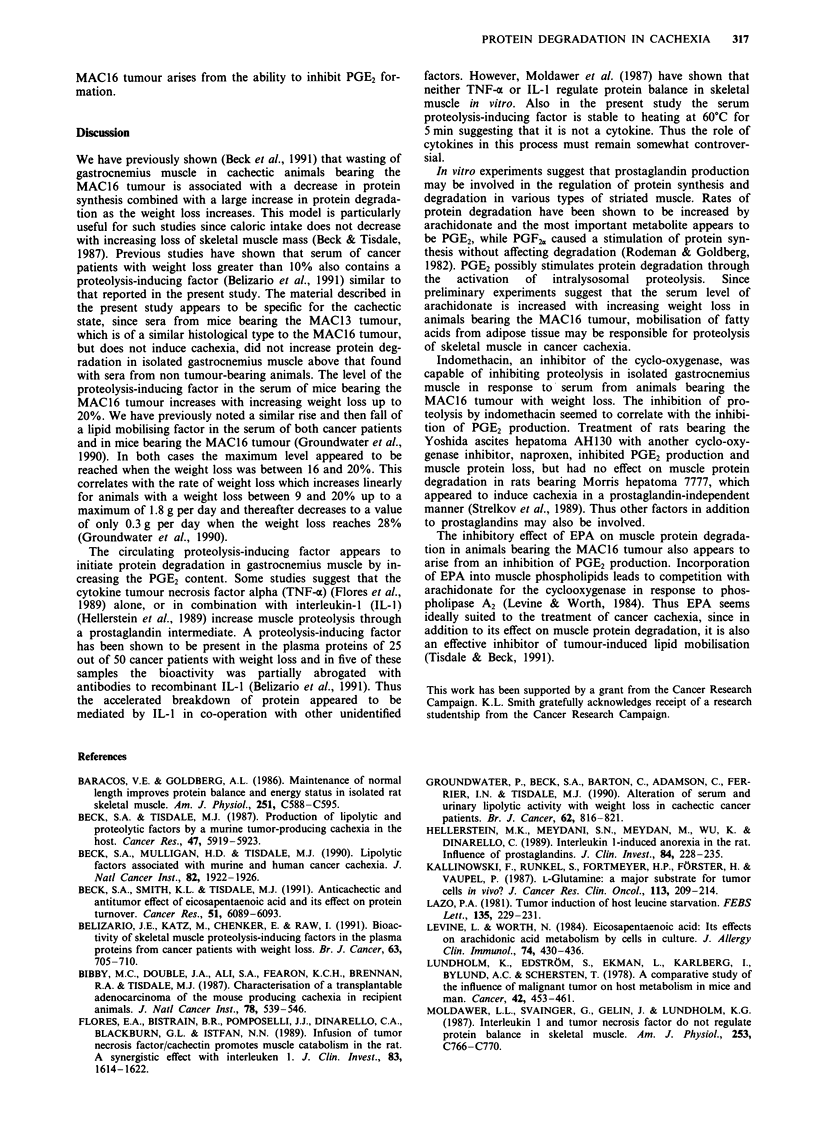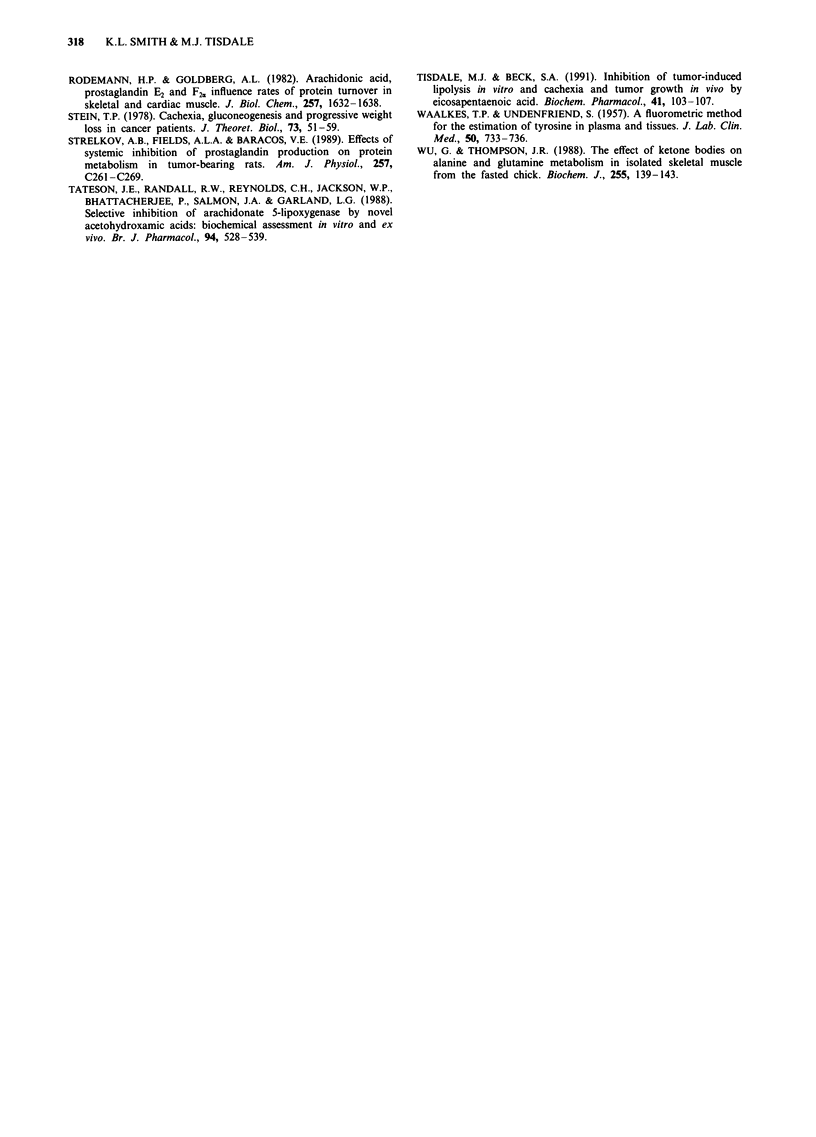# Mechanism of muscle protein degradation in cancer cachexia.

**DOI:** 10.1038/bjc.1993.334

**Published:** 1993-08

**Authors:** K. L. Smith, M. J. Tisdale

**Affiliations:** Pharmaceutical Sciences Institute, Aston University, Birmingham, UK.

## Abstract

Depletion of skeletal muscle mass in animals bearing an experimental model of cachexia, the MAC16 adenocarcinoma, occurs by a reduction in protein synthesis accompanied by a large increase in protein degradation. Serum from mice bearing the MAC16 tumour produced an increased protein degradation in isolated gastrocnemius muscle, as measured by tyrosine release, with a maximal effect occurring with serum from animals with a weight loss of between 11 and 20%. The response was specific to the cachectic state, since serum from mice bearing the MAC13 adenocarcinoma, which does not produce weight loss, did not increase tyrosine release from gastrocnemius muscle above that observed with serum from non tumour-bearing animals. The circulatory proteolysis-inducing factor was stable to heating at 60 degrees C for 5 min and was not inhibited by phenylmethylsulfonyl fluoride, suggesting that it was not a serine protease. The level of prostaglandin E2 (PGE2) in gastrocnemius muscle was significantly elevated after incubation with serum from cachectic mice bearing the MAC16 tumour. Both indomethacin and the polyunsaturated fatty acid eicosapentaenoic acid (EPA) inhibited the rise in muscle PGE2 content in response to serum from cachectic mice and also inhibited muscle protein degradation. These results suggest that muscle protein degradation in cancer cachexia is associated with a rise in PGE2 content.


Br. J. Cancer (1993), 68, 314–318 © Macmillan Press Ltd., 1993

### Mechanism of muscle protein degradation in cancer cachexia

K.L. Smith & M.J. Tisdale

*Cancer Research Campaign Experimental Chemotherapy Group, Pharmaceutical Sciences Institute, Aston University, Aston Triangle, Birmingham, B4 7ET, UK.*

**Summary** Depletion of skeletal muscle mass in animals bearing an experimental model of cachexia, the MAC16 adenocarcinoma, occurs by a reduction in protein synthesis accompanied by a large increase in protein degradation. Serum from mice bearing the MAC16 tumour produced an increased protein degradation in isolated gastrocnemius muscle, as measured by tyrosine release, with a maximal effect occurring with serum from animals with a weight loss of between 11 and 20%. The response was specific to the cachectic state, since serum from mice bearing the MAC13 adenocarcinoma, which does not produce weight loss, did not increase tyrosine release from gastrocnemius muscle above that observed with serum from non tumour-bearing animals. The circulatory proteolysis-inducing factor was stable to heating at 60°C for 5 min and was not inhibited by phenylmethylsulfonyl fluoride, suggesting that it was not a serine protease. The level of prostaglandin $E_2$ ($PGE_2$) in gastrocnemius muscle was significantly elevated after incubation with serum from cachectic mice bearing the MAC16 tumour. Both indomethacin and the polyunsaturated fatty acid eicosapentaenoic acid (EPA) inhibited the rise in muscle $PGE_2$ content in response to serum from cachectic mice and also inhibited muscle protein degradation. These results suggest that muscle protein degradation in cancer cachexia is associated with a rise in $PGE_2$ content.

Muscle wasting associated with cancer cachexia is an important complication in the management of the cancer patient and can lead to death through a depletion of cardiac and respiratory muscles. Maintenance of skeletal muscle mass is a balance between the rate of protein synthesis and the rate of protein degradation, and in mice bearing an experimental model of cachexia, the MAC16 colon adenocarcinoma, protein synthesis is reduced and protein degradation is increased with increasing weight loss (Beck *et al.*, 1991). This model is particularly attractive for studying changes in protein balance with the development of cachexia, since weight loss occurs without a reduction in food intake and reaches 30% when the tumour mass only represents 3% of the host body weight (Beck & Tisdale, 1987).

The relevance of protein degradation to the overall aietology of the tumour remains unknown, although an increased requirement for certain amino acids particularly leucine (Lazo, 1981) and glutamine (Kallinowski *et al.*, 1987) has been observed in the tumour-bearing state. Stein (1978) has attributed the abnormal gluconeogenesis seen in cancer patients to the avidity of the tumour for certain amino acids, which left the host with the problem of disposing of the remainder. Removal of certain amino acids by the tumour would lead to a depression of host protein synthesis since normal protein synthesis requires the full complement of amino acids.

The mechanism for the increased protein degradation in cancer cachexia remains unknown, although we have noted increased levels of a proteolysis-inducing factor in the serum of mice bearing the MAC16 adenocarcinoma (Beck & Tisdale, 1987). Such circulating factors may act to increase the level of lysosomal enzymes, such as cathepsin D, which may be involved in the intracellular breakdown of macromolecules. Thus Lundholm *et al.* (1978) have demonstrated an increased concentration of cathepsin D in skeletal muscle tissue from cancer patients and tumour-bearing mice.

The present study further investigates the mechanism for protein degradation in gastrocnemius muscle of mice bearing the MAC16 tumour with particular reference to the serum factor previously reported (Beck & Tisdale, 1987). In addition the effect of inhibitors on this process have been determined.

Correspondence: M.J. Tisdale.
Received 23 November 1992; and in revised form 26 March 1993.

### Materials and methods

### *Animals*

Pure strain female NMRI mice were obtained from our own breeding colony and were fed a rat and mouse breeding diet (Pilsbury Ltd., Birmingham, UK) and water *ad libitum.* Animals (average body weight 20 g) were transplanted with fragments of the MAC16 tumour into the flank by means of a trocar as previously described (Bibby *et al.*, 1987). Weight loss started to occur 10 to 12 days after transplantation when the tumours became palpable and animals were used with varying degrees of weight loss up to a maximum of 25 to 30% as agreed by the Coordinating Committee on Cancer Research of the United Kingdom for the welfare of animals with neoplasms. Blood was removed from animals by cardiac puncture under anesthesia using a mixture of halothane, oxygen and nitrous oxide between 9.30 and 10.30a.m. Blood samples were allowed to clot for 10 min at room temperature and serum was produced by centrifugation at 13,000 rpm for 5 min an a microfuge. Serum samples were stored at – 70°C until required.

### *Chemicals*

Indomethacin, prostaglandin $E_2$ ($PGE_2$), rabbit antisera to $PGE_2$ were purchased from Sigma Chemical Co., Poole, Dorset, United Kingdom. Eicosapentaenoic acid (EPA) (80%, expressed as a percentage of fatty acid methyl esters prepared) was kindly donated by Dr D. Horrobin, Scotia Pharmaceuticals Ltd., Guildford, Surrey, United Kingdom. BW A4C was kindly supplied by Dr L.G. Garland, Wellcome Research Laboratories, Beckenham, Kent, United Kingdom.

### *Measurement of protein degradation*

Female NMRI mice were killed by cervical dislocation and their gastrocnemius muscles were quickly ligated, dissected out and placed in ice-cold isotonic saline. For the experiment presented in Figure 6 animals were administered pure EPA (2 g per kg per day) orally for 5 days prior to the isolation of the gastrocnemius muscle. All animals were sacrificed between 9–10a.m. to minimise diurnal variation and were assured to be in the fed state. The muscles were then blotted, weighed and carefully tied via tendon ligatures (Wu & Thompson, 1988) to stainless steel incubation supports to prevent contraction, thus improving protein balance and energy status (Baracos & Goldberg, 1986). Protein degradation was measured by tyrosine release, since tyrosine rapidly equilibrates between intracellular pools and the medium and it is neither synthesised nor degraded. Muscles were preincubated in Dulbecco's minimal essential medium (DMEM) (3 ml) lacking phenol red and saturated with $O_2$:$CO_2$ (19:1) in the presence of serum (280 µl). After 30 min at 37°C the muscles were rinsed and incubated in Krebs-Henseleit bicarbonate buffer for a further 2 h. After the final 2 h incubation the buffer was removed, deproteinised with ice-cold 30% trichloroacetic acid (0.2 ml), centrifuged at 2800 *g* for 10 min and the supernatants were used for the measurement of tyrosine by a fluorimetric method (Waalkes & Undenfriend, 1957) at 570 nm on a Perkin-Elmer LS-5 luminescene spectrometer.

*Determination of $PGE_2$ levels in muscle samples*

Slices of gastrocnemius muscle were incubated in Krebs-Ringer bicarbonate buffer (2 ml) supplemented with glucose (1 mg $ml^{-1}$) and bovine serum albumin (1 mg $ml^{-1}$) in a shaking water bath at 37°C. Muscle preparations were incubated initially for 20 min under an atmosphere of 5% ($CO_2$, 95% $N_2$ and then for a further 15 min under an atmosphere of 5% $CO_2$, 95% $O_2$. At the end of the incubation period an aliquot (1 ml) of the surrounding buffer was removed, adjusted to pH 3 with 2 M HCl and extracted twice with ethyl acetate saturated with water (3 ml). The organic layer was removed, evaporated to dryness under a stream of nitrogen and dissolved in 1 ml of 0.025 M phosphate, pH 6.8, containing 0.01 M EDTA, 0.9% NaCl, 0.3% bovine gamma globulin, 0.005% triton X-100 and 0.05% sodium azide. The concentration of $PGE_2$ in the sample was determined using a radioimmunoassay procedure employing rabbit anti-$PGE_2$ antisera. [5,6,8,11,12,14,15(N)-$^3$H] Prostaglandin $E_2$ (specific activity 150 Ci $mmol^{-1}$) (Amersham International, Amersham, UK) was diluted to give a concentration of 4.26 nCi/assay. Bound and unbound material was separated using dextran coated charcoal and separated by centrifugation.

### Results

We have previously shown that loss of skeletal muscle protein in mice bearing the MAC16 adenocarcinoma arises from a depression of protein synthesis accompanied by a massive increase in protein degradation, which increases with increasing weight loss (Beck *et al.*, 1991). Using the isolated gastrocnemius muscle model an increased protein degradation as measured by tyrosine release, can be produced by incubation with serum from mice bearing the MAC16 tumour (Figure 1). Increasing weight loss produces an increased degradation activity up to a weight loss of 20%, after which the level decreases to a value not significantly different from that found in animals without weight loss.

**Figure 1** Effect of serum from mice bearing the MAC16 tumour and with progressive weight loss on tyrosine release from gastrocnemius muscle. Serum (280 µl i.e. 7% of assay volume) was added to freshly isolated gastrocnemius muscle isolated from non tumour-bearing animals and the tyrosine released during a 2 h incubation was determined as described in Methods. Each bar represents the mean ± s.e.m. of four animals. Differences were determined by one-way analysis of variance as $^*P<0.05$ and $^{**}P<0.01$ from non tumour-bearing animals.

This effect appears to be specific to serum from cachectic animals since serum from mice bearing a closely related tumour, MAC13, which does not induce cachexia, did not increase tyrosine release from gastrocnemius muscle above that observed with non tumour-bearing animals (Figure 2). The proteolysis-inducing factor in the serum from animals bearing the MAC16 tumour is stable to heating at 60°C for 5 min (Figure 2) and is not inhibited by 1 mM phenylmethylsulfonyl fluoride, suggesting that it is not a serine protease. Inhibition could, however, be achieved by the addition of the cycloxoygenase inhibitor indomethacin (0.2 mM). There was a decrease by both the polyunsaturated fatty acid EPA (0.5 mM) and the lipoxygenase inhibitor BWA4C (Tateson *et al.*, 1988) at high concentrations (1.77 mM) although the values were still significantly elevated compared to the control. Protein degradation in isolated gastrocnemius muscle could not be induced by the purified lipid mobilising factor produced by the MAC16 tumour (Beck *et al.*, 1990). These results suggest that serum from cachectic animals bearing the MAC16 tumour acts to initiate protein degradation in skeletal muscle through the intermediacy of a prostaglandin intermediate.

This view is substantiated by the significant elevation in gastrocnemius muscle $PGE_2$ content after incubation with serum from cachectic mice bearing the MAC16 tumour, when compared with that observed with serum from non tumour-bearing animals (Figure 3). The effect appeared to arise from a stimulation of $PGE_2$ production by the gastrocnemius muscle, since the $PGE_2$ concentration of serum from cachectic animals bearing the MAC16 tumour (111 pg $ml^{-1}$) was lower than that found in non tumour-bearing animals (147 pg $ml^{-1}$). Indomethacin reduced both tyrosine release from gastrocnemius muscle in response to serum from cachectic animals and the subsequent elevation in $PGE_2$ content in a dose-related manner (Figure 4). A large (66%) reduction in muscle $PGE_2$ content was required before a significant reduction in muscle proteolysis was observed.

**Figure 2** Effect of serum from non tumour-bearing animals (1), animals bearing the MAC13 tumour (2), the MAC16 tumour from mice with 11–16% weight loss (3–8) or a partially purified lipid mobilising factor (9) on tyrosine release from isolated gastrocnemius muscle. Serum from animals bearing the MAC16 tumour was used as such (3), heated to 60°C for 5 min (4), treated with phenylmethylsulfonyl fluoride (1 mM) (5), indomethacin (0.2 mM) (6), EPA (0.5 mM) (7) or BWA4C (1.77 mM) (8). Each bar represents the mean ± s.e.m. of three animals. Differences were determined by one-way analysis of variance as $^*P<0.05$ and $^{**}P<0.01$ compared to group 1.

**Figure 3** Effect of serum from non tumour-bearing animals (closed box) and animals bearing the MAC16 tumour and with weight loss 11–15% (hatched box) on the $PGE_2$ content of isolated gastrocnemius muscle. Each bar represents the mean ± s.e.m. of six animals. Differences were determined by Student's *t*-test as $^{***}P<0.001$ from muscles treated with serum from non tumour-bearing animals.

We have recently reported that eicosapentaenoic acid (EPA) is an effective inhibitor of the weight loss in animals bearing MAC16 tumour (Beck *et al.*, 1991). Maintenance of skeletal muscle mass by EPA in animals bearing the MAC16 tumour was found to arise from a significant reduction (60%) in muscle protein degradation without an effect on protein synthesis. The inhibitory effect of EPA on muscle protein degradation may result from its ability to inhibit $PGE_2$ synthesis. The results presented in Figure 5 show that addition of EPA directly to the *in vitro* gastrocnemius muscle preparation caused a dose-related reduction in both tyrosine release and $PGE_2$ production in response to serum from

**Figure 5** Effect of EPA on the $PGE_2$ content (hatched box) and tyrosine release (closed box) from isolated gastrocnemius muscle in response to serum from animals bearing the MAC16 tumour and with weight loss 11–15%. Each bar represents the mean ± s.e.m. of three animals. Differences were determined by one-way analysis of variance as $^{*}P<0.05$ and $^{**}P<0.01$ compared to no addition of EPA.

cachectic animals. However, high concentrations (500 μM) of EPA were required to produce a significant reduction in muscle $PGE_2$ content and tyrosine release.

The low effectiveness of EPA in this *in vitro* assay may be due to poor incorporation of the fatty acid into muscle lipids, since pre-treatment of animals with EPA ($2\,g\,kg^{-1}$) for 5 days prior to the assay was much more effective in inhibiting both tyrosine release and the $PGE_2$ content of the isolated gastrocnemius muscle in response to serum from cachectic mice bearing the MAC16 tumour (Figure 6). Using muscles from animals not pre-treated with EPA there was a significant ($P<0.01$) increase in tyrosine release and $PGE_2$ content in response to serum from cachectic mice when compared with that observed with non tumour-bearing animals. However, in gastrocnemius muscle isolated from non tumour-bearing animals previously pre-treated with EPA, there was a significant reduction ($P<0.001$) in both protein degradation, as measured by tyrosine release and $PGE_2$ content. This confirms that the pre-treatment of the donor muscle with EPA reduces protein degradation to levels seen in muscles produced by serum from non tumour-bearing animals. These results suggest that the maintenance of skeletal muscle mass by EPA in cachectic animals bearing the

**Figure 4** Effect of indomethacin on tyrosine release from isolated gastrocnemius muscle (closed boxes) and $PGE_2$ content (hatched boxes) after a 2 h incubation. Results are expressed as mean ± s.e.m. for 12 determinations per group. Differences were determined by one-way analysis of variance as $^{*}P<0.05$ and $^{**}P<0.01$ compared to muscles incubated in the absence of indomethacin.

**Figure 6** Effect of treatment of mice with EPA (2.0 g kg day) for 5 days by gavage on the response of isolated gastrocnemius muscle to induction of protein degradation as measured by tyrosine release (solid boxes) and muscle $PGE_2$ content (hatched boxes) in response to serum (280 μl) from cachectic animals bearing the MAC16 tumour (weight loss 10–15%). Differences were determined by Student's *t*-test as $^{***}P<0.001$ from animals not pre-treated with EPA.

MAC16 tumour arises from the ability to inhibit $PGE_2$ formation.

### Discussion

We have previously shown (Beck *et al.*, 1991) that wasting of gastrocnemius muscle in cachectic animals bearing the MAC16 tumour is associated with a decrease in protein synthesis combined with a large increase in protein degradation as the weight loss increases. This model is particularly useful for such studies since caloric intake does not decrease with increasing loss of skeletal muscle mass (Beck & Tisdale, 1987). Previous studies have shown that serum of cancer patients with weight loss greater than 10% also contains a proteolysis-inducing factor (Belizario *et al.*, 1991) similar to that reported in the present study. The material described in the present study appears to be specific for the cachectic state, since sera from mice bearing the MAC13 tumour, which is of a similar histological type to the MAC16 tumour, but does not induce cachexia, did not increase protein degradation in isolated gastrocnemius muscle above that found with sera from non tumour-bearing animals. The level of the proteolysis-inducing factor in the serum of mice bearing the MAC16 tumour increases with increasing weight loss up to 20%. We have previously noted a similar rise and then fall of a lipid mobilising factor in the serum of both cancer patients and in mice bearing the MAC16 tumour (Groundwater *et al.*, 1990). In both cases the maximum level appeared to be reached when the weight loss was between 16 and 20%. This correlates with the rate of weight loss which increases linearly for animals with a weight loss between 9 and 20% up to a maximum of 1.8 g per day and thereafter decreases to a value of only 0.3 g per day when the weight loss reaches 28% (Groundwater *et al.*, 1990).

The circulating proteolysis-inducing factor appears to initiate protein degradation in gastrocnemius muscle by increasing the $PGE_2$ content. Some studies suggest that the cytokine tumour necrosis factor alpha (TNF-$\alpha$) (Flores *et al.*, 1989) alone, or in combination with interleukin-1 (IL-1) (Hellerstein *et al.*, 1989) increase muscle proteolysis through a prostaglandin intermediate. A proteolysis-inducing factor has been shown to be present in the plasma proteins of 25 out of 50 cancer patients with weight loss and in five of these samples the bioactivity was partially abrogated with antibodies to recombinant IL-1 (Belizario *et al.*, 1991). Thus the accelerated breakdown of protein appeared to be mediated by IL-1 in co-operation with other unidentified factors. However, Moldawer *et al.* (1987) have shown that neither TNF-$\alpha$ or IL-1 regulate protein balance in skeletal muscle *in vitro*. Also in the present study the serum proteolysis-inducing factor is stable to heating at 60°C for 5 min suggesting that it is not a cytokine. Thus the role of cytokines in this process must remain somewhat controversial.

*In vitro* experiments suggest that prostaglandin production may be involved in the regulation of protein synthesis and degradation in various types of striated muscle. Rates of protein degradation have been shown to be increased by arachidonate and the most important metabolite appears to be $PGE_2$, while $PGF_{2\alpha}$ caused a stimulation of protein synthesis without affecting degradation (Rodeman & Goldberg, 1982). $PGE_2$ possibly stimulates protein degradation through the activation of intralysosomal proteolysis. Since preliminary experiments suggest that the serum level of arachidonate is increased with increasing weight loss in animals bearing the MAC16 tumour, mobilisation of fatty acids from adipose tissue may be responsible for proteolysis of skeletal muscle in cancer cachexia.

Indomethacin, an inhibitor of the cyclo-oxygenase, was capable of inhibiting proteolysis in isolated gastrocnemius muscle in response to serum from animals bearing the MAC16 tumour with weight loss. The inhibition of proteolysis by indomethacin seemed to correlate with the inhibition of $PGE_2$ production. Treatment of rats bearing the Yoshida ascites hepatoma AH130 with another cyclo-oxygenase inhibitor, naproxen, inhibited $PGE_2$ production and muscle protein loss, but had no effect on muscle protein degradation in rats bearing Morris hepatoma 7777, which appeared to induce cachexia in a prostaglandin-independent manner (Strelkov *et al.*, 1989). Thus other factors in addition to prostaglandins may also be involved.

The inhibitory effect of EPA on muscle protein degradation in animals bearing the MAC16 tumour also appears to arise from an inhibition of $PGE_2$ production. Incorporation of EPA into muscle phospholipids leads to competition with arachidonate for the cyclooxygenase in response to phospholipase $A_2$ (Levine & Worth, 1984). Thus EPA seems ideally suited to the treatment of cancer cachexia, since in addition to its effect on muscle protein degradation, it is also an effective inhibitor of tumour-induced lipid mobilisation (Tisdale & Beck, 1991).

This work has been supported by a grant from the Cancer Research Campaign. K.L. Smith gratefully acknowledges receipt of a research studentship from the Cancer Research Campaign.

### References

BARACOS, V.E. & GOLDBERG, A.L. (1986). Maintenance of normal length improves protein balance and energy status in isolated rat skeletal muscle. *Am. J. Physiol.*, **251**, C588–C595.

BECK, S.A. & TISDALE, M.J. (1987). Production of lipolytic and proteolytic factors by a murine tumor-producing cachexia in the host. *Cancer Res.*, **47**, 5919–5923.

BECK, S.A., MULLIGAN, H.D. & TISDALE, M.J. (1990). Lipolytic factors associated with murine and human cancer cachexia. *J. Natl Cancer Inst.*, **82**, 1922–1926.

BECK, S.A., SMITH, K.L. & TISDALE, M.J. (1991). Anticachectic and antitumor effect of eicosapentaenoic acid and its effect on protein turnover. *Cancer Res.*, **51**, 6089–6093.

BELIZARIO, J.E., KATZ, M., CHENKER, E. & RAW, I. (1991). Bioactivity of skeletal muscle proteolysis-inducing factors in the plasma proteins from cancer patients with weight loss. *Br. J. Cancer*, **63**, 705–710.

BIBBY, M.C., DOUBLE, J.A., ALI, S.A., FEARON, K.C.H., BRENNAN, R.A. & TISDALE, M.J. (1987). Characterisation of a transplantable adenocarcinoma of the mouse producing cachexia in recipient animals. *J. Natl Cancer Inst.*, **78**, 539–546.

FLORES, E.A., BISTRAIN, B.R., POMPOSELLI, J.J., DINARELLO, C.A., BLACKBURN, G.L. & ISTFAN, N.N. (1989). Infusion of tumor necrosis factor/cachectin promotes muscle catabolism in the rat. A synergistic effect with interleuken 1. *J. Clin. Invest.*, **83**, 1614–1622.

GROUNDWATER, P., BECK, S.A., BARTON, C., ADAMSON, C., FERRIER, I.N. & TISDALE, M.J. (1990). Alteration of serum and urinary lipolytic activity with weight loss in cachectic cancer patients. *Br. J. Cancer*, **62**, 816–821.

HELLERSTEIN, M.K., MEYDANI, S.N., MEYDAN, M., WU, K. & DINARELLO, C. (1989). Interleukin 1-induced anorexia in the rat. Influence of prostaglandins. *J. Clin. Invest.*, **84**, 228–235.

KALLINOWSKI, F., RUNKEL, S., FORTMEYER, H.P., FÖRSTER, H. & VAUPEL, P. (1987). L-Glutamine: a major substrate for tumor cells *in vivo*? *J. Cancer Res. Clin. Oncol.*, **113**, 209–214.

LAZO, P.A. (1981). Tumor induction of host leucine starvation. *FEBS Lett.*, **135**, 229–231.

LEVINE, L. & WORTH, N. (1984). Eicosapentaenoic acid: Its effects on arachidonic acid metabolism by cells in culture. *J. Allergy Clin. Immunol.*, **74**, 430–436.

LUNDHOLM, K., EDSTRÖM, S., EKMAN, L., KARLBERG, I., BYLUND, A.C. & SCHERSTEN, T. (1978). A comparative study of the influence of malignant tumor on host metabolism in mice and man. *Cancer*, **42**, 453–461.

MOLDAWER, L.L., SVAINGER, G., GELIN, J. & LUNDHOLM, K.G. (1987). Interleukin 1 and tumor necrosis factor do not regulate protein balance in skeletal muscle. *Am. J. Physiol.*, **253**, C766–C770.

RODEMANN, H.P. & GOLDBERG, A.L. (1982). Arachidonic acid, prostaglandin $E_2$ and $F_{2\alpha}$ influence rates of protein turnover in skeletal and cardiac muscle. *J. Biol. Chem.*, **257**, 1632–1638.

STEIN, T.P. (1978). Cachexia, gluconeogenesis and progressive weight loss in cancer patients. *J. Theoret. Biol.*, **73**, 51–59.

STRELKOV, A.B., FIELDS, A.L.A. & BARACOS, V.E. (1989). Effects of systemic inhibition of prostaglandin production on protein metabolism in tumor-bearing rats. *Am. J. Physiol.*, **257**, C261–C269.

TATESON, J.E., RANDALL, R.W., REYNOLDS, C.H., JACKSON, W.P., BHATTACHERJEE, P., SALMON, J.A. & GARLAND, L.G. (1988). Selective inhibition of arachidonate 5-lipoxygenase by novel acetohydroxamic acids: biochemical assessment *in vitro* and *ex vivo*. *Br. J. Pharmacol.*, **94**, 528–539.

TISDALE, M.J. & BECK, S.A. (1991). Inhibition of tumor-induced lipolysis *in vitro* and cachexia and tumor growth *in vivo* by eicosapentaenoic acid. *Biochem. Pharmacol.*, **41**, 103–107.

WAALKES, T.P. & UNDENFRIEND, S. (1957). A fluorometric method for the estimation of tyrosine in plasma and tissues. *J. Lab. Clin. Med.*, **50**, 733–736.

WU, G. & THOMPSON, J.R. (1988). The effect of ketone bodies on alanine and glutamine metabolism in isolated skeletal muscle from the fasted chick. *Biochem. J.*, **255**, 139–143.